# Comparison of ultrasound-guided serratus posterior superior intercostal plane block and thoracic paravertebral block for postoperative analgesia after breast surgery: a prospective randomized noninferiority trial

**DOI:** 10.55730/1300-0144.6201

**Published:** 2026-03-24

**Authors:** Mustafa Sırrı KOTANOĞLU, Musa ZENGİN, Onur KÜÇÜK, Atakan SEZGİ, İbrahim TOPCU, Aras METİN, Zeynep ERSOY, Jülide ERGİL

**Affiliations:** 1 Department of Anesthesiology and Reanimation, Ankara Etlik City Hospital, University of Health Sciences, Ankara, Turkiye; 2 Department of Anesthesiology and Reanimation, Faculty of Medicine, University of Dokuz Eylül, İzmir, Turkiye

**Keywords:** Serratus posterior superior intercostal plane block, thoracic paravertebral block, noninferiority trial, breast surgery, postoperative analgesia, regional anesthesia

## Abstract

**Background/aim:**

This noninferiority trial aimed to evaluate whether the serratus posterior superior intercostal plane block (SPSIPB) provides noninferior postoperative analgesia compared with the thoracic paravertebral block (TPVB) after unilateral mastectomy.

**Materials and methods:**

This prospective, randomized, assessor-blinded, parallel-group noninferiority trial enrolled 60 female patients scheduled for elective unilateral mastectomy. Patients received either ultrasound-guided SPSIPB (n = 30) or TPVB (n = 30) with 30 mL of 0.25% bupivacaine before induction. The primary outcome was visual analogue scale (VAS) pain scores (0–100 mm) at rest and during coughing over 24 h. Noninferiority was assessed using the Hodges–Lehmann median difference with 95% confidence intervals (CIs) and a prespecified margin of 13 mm, corresponding to the validated minimal clinically important difference (MCID). Secondary outcomes included opioid consumption, area under the curve (AUC) for cumulative pain burden, and patient satisfaction.

**Results:**

All 60 patients completed the study. At the first postoperative hour, TPVB provided lower VAS scores at rest (median 10.0 vs. 23.0 mm, 95% CI of difference: 0 to 19). For resting VAS, noninferiority was demonstrated at 0 h, 4 h, and 24 h (upper bounds: 11.5 mm, 12.5 mm, and 5.0 mm). The 24-h AUC for resting VAS was comparable between groups (426 mm/h vs. 426.5 mm/h, p = 0.652). SPSIPB produced significantly lower tramadol consumption in the 12-to-24-h interval (median 0 vs. 50 mg, p < 0.001). However, total opioid consumption over the 24 h was comparable (p = 0.070). No block-related complications occurred in either group.

**Conclusion:**

Noninferiority of SPSIPB to TPVB was demonstrated for resting pain scores at the majority of postoperative time points after unilateral mastectomy. TPVB provided a transient early-phase analgesic advantage at 1 h and 2 h, while SPSIPB was associated with late-phase opioid sparing. The comparable cumulative pain burden across 24 h suggests that SPSIPB may serve as a periparavertebral alternative to TPVB when sustained analgesia and opioid reduction are clinical priorities.

## 1. Introduction

Breast surgery is frequently associated with moderate-to-severe acute postoperative pain, which may adversely affect early recovery and functional outcomes [1]. Persistent pain following breast cancer surgery develops in up to 47% of patients, and acute postoperative pain severity has been identified as one of the strongest predictors of chronicity [2,3]. Among women who develop chronic postsurgical pain, 13% report severe symptoms that interfere with daily activities [2]. These data emphasize the clinical need for effective perioperative analgesia strategies in this population.

Multimodal analgesia incorporating regional anesthesia has become the standard approach to pain management after breast surgery [1,4]. Thoracic paravertebral block (TPVB) provides unilateral segmental analgesia by depositing local anesthetic adjacent to the spinal nerve roots, and three recent network metaanalyses encompassing more than 16,000 patients across 241 randomized controlled trials (RCTs) have consistently ranked TPVB among the most effective techniques for this indication [4–6]. Singh et al. reported the highest Surface Under the Cumulative Ranking Curve score for continuous TPVB (0.83) among 11 techniques in 5686 patients [4], while De Cassai et al. found that no single technique was clearly superior across all outcomes in 62 RCTs [5].

TPVB is technically demanding, however, and carries risks related to its anatomical proximity to the pleura and neuraxis, including pneumothorax, vascular injury, and inadvertent neuraxial spread [1]. These concerns have driven interest in alternative interfascial plane blocks that may offer comparable analgesia with an easier ultrasound-guided technique and fewer complications. The erector spinae plane block (ESPB) was the first widely studied alternative, but randomized comparisons have yielded inconsistent results; Swisher et al. rejected noninferiority of ESPB to paravertebral block in 100 patients undergoing breast surgery [7], and metaanalytic data suggest that ESPB provides variable paravertebral spread in only 30–40% of cases [8,9]. A recent metaanalysis with trial sequential analysis further confirmed that TPVB is not superior to the interpectoral-pectoserratus (PECS-II) block for 24-h opioid consumption after breast surgery, leaving the question of the optimal technique unresolved [10].

The serratus posterior superior intercostal plane block (SPSIPB) is a recently described periparavertebral technique in which local anesthetic is deposited between the serratus posterior superior muscle and the intercostal muscles [11]. Cadaveric evaluation by Tulgar et al. demonstrated dye spread from C7 to T7, with staining of dorsal rami and lateral cutaneous branches of intercostal nerves across multiple thoracic levels [11]. Early clinical reports confirmed low pain scores and minimal opioid requirements after SPSIPB in breast and thoracic surgery [12–14]. The first RCT of SPSIPB in breast surgery, conducted by Köksal et al. in 60 patients, demonstrated a 45% reduction in 24-h tramadol consumption compared with a no-block control group [15]. Arık et al. subsequently reported that SPSIPB produced lower tramadol consumption than ESPB with equivalent pain scores in 50 patients undergoing breast surgery [16]. However, no randomized trial has directly compared SPSIPB with TPVB in breast surgery using a noninferiority framework.

Given the absence of noninferiority evidence comparing these two techniques in breast surgery, a randomized comparison with a clinically meaningful margin is warranted. The aim of this study was to evaluate the noninferiority of ultrasound-guided SPSIPB compared with TPVB for postoperative pain control in patients undergoing unilateral mastectomy. We hypothesized that SPSIPB would be noninferior to TPVB with respect to postoperative VAS pain scores over 24 h, defined by a prespecified noninferiority margin of 13 mm [17].

## 2. Materials and methods

### 2.1. Study design

This prospective, randomized, assessor-blinded, parallel-group, noninferiority clinical trial was conducted at Ankara Etlik City Hospital after approval by the Ankara Etlik City Hospital Scientific Research Evaluation and Ethics Board (Approval No.: AESH-EK-2025-07, Date: 15 January 2025). The study was registered at ClinicalTrials.gov prior to patient enrollment (NCT06789146, February 2025). Written informed consent was obtained from all participants. The trial was conducted in accordance with the Declaration of Helsinki (2013 amendment) and reported in accordance with the CONSORT guidelines for noninferiority randomized clinical trials.

### 2.2. Inclusion and exclusion criteria

The study was conducted between February 2025 and February 2026. Female patients aged 18 to 80 y scheduled for elective unilateral mastectomy under general anesthesia were assessed for eligibility. Inclusion criteria were an American Society of Anesthesiologists (ASA) physical status of I–III and a body mass index (BMI) between 18 and 30 kg/m^2^.

Exclusion criteria included refusal to participate, ASA physical status IV or higher, known coagulopathy or bleeding diathesis, infection at the block site, allergy to local anesthetics, BMI below 18 kg/m^2^ or above 30 kg/m^2^, severe hepatic or renal dysfunction, and inability to understand or use the VAS.

### 2.3. Randomization and blinding

Eligible patients were randomly allocated in a 1:1 ratio to either the SPSIPB group or the TPVB group using a computer-generated randomization sequence. Allocation concealment was ensured by sealed, opaque envelopes opened immediately before block performance. The study was assessor-blinded: the anesthesiologist performing postoperative pain assessments and data collection was not present during block performance and was unaware of group assignment. Patients were not blinded to their allocation, and the operator performing the block was necessarily aware of the assigned technique.

### 2.4. Block procedures

After admission to the dedicated block area, routine monitoring was applied (noninvasive arterial blood pressure, electrocardiography, heart rate, and peripheral oxygen saturation). Venous access was obtained with a 20-gauge intravenous cannula, and procedural sedation was provided with intravenous midazolam (0.03 mg/kg). All blocks were performed by the same anesthesiologist with extensive experience in ultrasound-guided regional anesthesia. The onset of the block procedure was defined as the time at which the ultrasound transducer first contacted the patient’s skin; procedure duration was recorded from needle insertion to needle removal. A high-frequency linear ultrasound transducer (6–15 MHz) and a 22-gauge, 80-mm block needle (Echoplex, Vygon, Ecouen, France) were used. All blocks were performed approximately 20 min before the induction of general anesthesia. Local anesthetic was injected incrementally with intermittent aspiration. In both groups, 30 mL of 0.25% bupivacaine was administered. Dermatomal sensory testing was not performed, as blocks were administered immediately before the induction of general anesthesia in all patients.

### 2.5. Serratus posterior superior intercostal plane block

Patients in the SPSIPB group were positioned in the lateral decubitus position with the surgical side uppermost. A high-frequency linear ultrasound transducer was placed medial to the scapula at the level of the third rib (corresponding to the T3 vertebral level) to identify the trapezius, rhomboid muscles, serratus posterior superior muscle, and the underlying intercostal muscles [11]. Using an in-plane approach, the block needle was advanced into the fascial plane between the serratus posterior superior muscle and the intercostal muscles. After negative aspiration, local anesthetic was injected incrementally, and the spread within the target interfascial plane was confirmed by real-time ultrasound imaging.

### 2.6. Thoracic paravertebral block

Patients in the TPVB group were positioned in the lateral decubitus position with the surgical side uppermost. The ultrasound transducer was placed in a parasagittal orientation to visualize the transverse process (T4), superior costotransverse ligament, pleura, and paravertebral space at the target thoracic level. Using an in-plane technique, the needle was advanced through the superior costotransverse ligament into the paravertebral space. After negative aspiration, local anesthetic was administered incrementally with sonographic confirmation of paravertebral spread.

### 2.7. Intraoperative and postoperative management

After the block was completed, patients were transferred to the operating theatre. General anesthesia was induced with propofol (2 mg/kg), fentanyl (1.5 mcg/kg), and vecuronium (0.1 mg/kg). Tracheal intubation was performed with an appropriately sized endotracheal tube. Anesthesia was maintained with sevoflurane (1–1.5%) in a 50:50 oxygen–air mixture, with a continuous remifentanil infusion (0.01–0.20 mcg/kg/min) titrated to hemodynamic parameters. Thirty min before the end of surgery, intravenous dexketoprofen (50 mg), tramadol (100 mg), and ondansetron (0.1 mg/kg) were administered to all patients. Neuromuscular blockade was reversed with neostigmine.

All patients received a standardized multimodal analgesic regimen postoperatively: paracetamol 1 g every 8 h, dexketoprofen 50 mg twice daily, and tramadol via patient-controlled analgesia (PCA). PCA settings were as follows: tramadol 5 mg/mL concentration, 20 mg bolus dose, 20-min lockout interval, 200 mg maximum in 4 h, and no background infusion. Postoperative pain was assessed by a blinded anesthesiologist using a 100-mm VAS (0 = no pain, 100 = worst pain imaginable) at rest and during coughing at 0 h, 1 h, 2 h, 4 h, 12 h, and 24 h postoperatively. Intravenous morphine (3 mg) was administered as rescue analgesia when resting VAS exceeded 40 mm. Total rescue morphine was converted to tramadol equivalent (morphine mg × 10 = tramadol mg) and added to PCA consumption for analysis.

### 2.8. Outcome measurements

The primary outcome was postoperative VAS pain scores at rest and during coughing measured at six time points (0 h, 1 h, 2 h, 4 h, 12 h, and 24 h). The noninferiority of SPSIPB to TPVB was assessed at each time point using the prespecified margin of 13 mm, corresponding to the validated minimal clinically important difference (MCID) on the 100-mm VAS [17].

Secondary outcomes included intraoperative remifentanil consumption, total tramadol and morphine consumption during the first 24 postoperative hours, the area under the curve (AUC) for cumulative 24-h pain burden calculated by the trapezoidal rule, pain reduction slopes, rescue analgesia requirements, postoperative nausea and vomiting (PONV), and patient satisfaction assessed by a five-point Likert scale at 24 h.

### 2.9. Sample size

The sample size was calculated for the primary outcome of VAS pain scores using a noninferiority margin of 13 mm, which corresponds to the MCID prospectively validated by Gallagher et al. for the 100-mm VAS [95% confidence intervals (CIs): 10–16 mm] [17] and is consistent with the margin adopted in prior SPSIPB trials [16,18] and postoperative pain studies [19]. Based on data from a prior randomized trial at our institution comparing SPSIPB with ESPB in breast surgery, the expected standard deviation for VAS scores was 15 mm [16]. Using a one-sided alpha of 0.025 (equivalent to two-sided 0.05) and 80% power, a minimum of 21 patients per group was required (Cohen’s d = 13/15 = 0.867). Accounting for a potential dropout rate of 10%, the target enrollment was set at 24 patients per group (48 total). We enrolled 30 patients per group (60 total) to provide an adequate safety margin against unforeseen protocol deviations. The calculation was performed using G*Power software (version 3.1, Heinrich-Heine-Universität Düsseldorf, Germany).

### 2.10. Statistical analyses

All statistical analyses were conducted using Jamovi software (Version 2.3.28; Sydney, Australia). Continuous variables were tested for normality using the Shapiro–Wilk test and for homogeneity of variances using Levene’s test. Normally distributed variables are presented as mean ± standard deviation (SD); nonnormally distributed variables as median [interquartile range (IQR)]. Categorical variables are presented as counts and percentages. Between-group comparisons used an independent samples Student’s t-test for normally distributed data and the Mann–Whitney *U* test for nonnormally distributed data. Categorical variables were compared using Pearson’s chi-square test or Fisher’s exact test when the expected cell frequency was less than five. Within-group changes in VAS scores over time were analyzed using the Friedman test, with post hoc Wilcoxon signed-rank tests and the Bonferroni correction for multiple comparisons. Between-group differences in pain trajectories were assessed by comparing delta values (changes from baseline) using the Mann–Whitney *U* test.

The noninferiority of SPSIPB to TPVB was assessed using the Hodges–Lehmann estimator of the median difference with a bootstrap 95% CI (1000 iterations). Noninferiority was declared if the upper bound of the 95% CI for the median difference (SPSIPB minus TPVB) did not exceed the prespecified margin of +13 mm for VAS scores [17]. The AUC was calculated using the trapezoidal rule with unequal time intervals. Pain reduction slopes were calculated for consecutive time intervals. Because no patients were lost to follow-up or excluded after randomization, the intention-to-treat and per-protocol populations were identical (n = 30 per group); results are therefore reported for this single analysis set. A two-sided p-value below 0.05 was considered statistically significant for secondary outcomes.

All six postoperative VAS assessment time points were prespecified in the study protocol prior to enrollment. Between-group comparisons at these time points were therefore considered confirmatory, and no additional multiplicity correction was applied. The Friedman test, as a nonparametric repeated-measures approach, addressed within-group temporal multiplicity through Bonferroni-corrected post hoc comparisons. Because VAS scores were nonnormally distributed at all time points, as indicated by Shapiro–Wilk testing, a linear mixed-effects model was not applied; a nonparametric framework was considered more appropriate for the skewed pain data. The 24-h AUC provided an integrated summary of the pain trajectory across all time points, reducing dependence on individual comparisons.

## 3. Results

**Figure 1:**
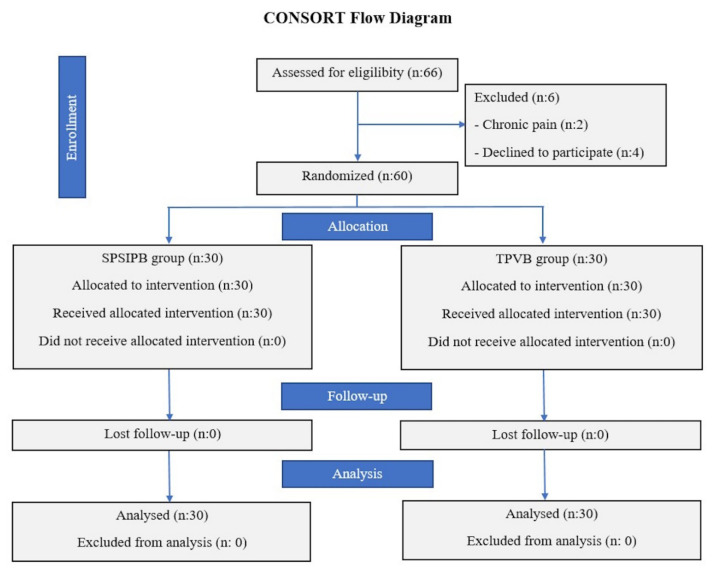
Flow chart of the patients. SPSIPB: serratus posterior superior intercostal plane block, TPVB: thoracic paravertebral block.

A total of 66 female patients undergoing unilateral mastectomy were evaluated, and 60 patients who met the inclusion criteria were randomly assigned to two groups: 30 to the SPSIPB group and 30 to the TPVB group. No patients were lost to follow-up or excluded from analysis (Figure 1). Demographic and clinical characteristics were comparable between groups (Table 1), with no statistically significant differences in age, BMI, ASA physical status, comorbidities, duration of surgery, or intraoperative remifentanil consumption (p > 0.05 for all comparisons).

**Figure 2:**
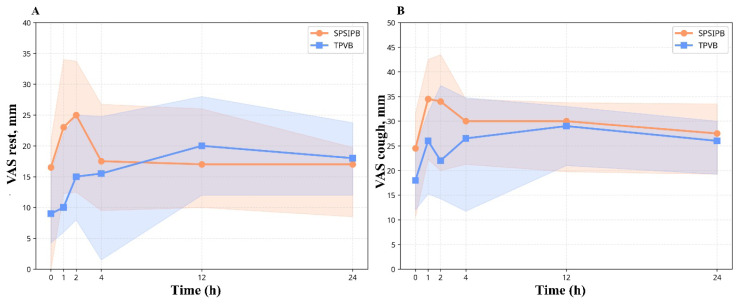
Postoperative 24-h VAS score trends (A: VAS at rest; B: VAS during coughing; shaded areas represent the IQR for each group.). SPSIPB: serratus posterior duperior intercostal plane block, TPVB: thoracic paravertebral block, VAS: Visual analogue scale.

Postoperative VAS scores at rest are presented in Table 2 and Figure 2. At the first postoperative hour, the TPVB group had lower median VAS scores at rest compared with the SPSIPB group [10 (IQR 6–22)] vs. 23 (IQR 12.2–34); Hodges–Lehmann median difference 13 mm, 95% CI: 0 to 19; p = 0.006). The change in pain from baseline to 1 h differed between groups: the SPSIPB group experienced a median increase of +6.5 mm, while the TPVB group increased by +1.0 mm (p = 0.024). No statistically significant between-group differences were observed at 0 h, 2 h, 4 h, 12 h, or 24 h (p > 0.05).

VAS scores during coughing are presented in Table 2 and Figure 2. At the first postoperative hour, the TPVB group had lower median VAS cough scores [26 (IQR 15.2–31.8) vs. 34.5 (IQR 22.2–42.5); median difference 8.5 mm, 95% CI: −4 to 19.5; p = 0.030]. No statistically significant between-group differences were observed at other time points (p > 0.05).

The Friedman test showed significant changes in VAS rest scores over time for both the SPSIPB group [chi-square (5) = 43.48, p < .001] and the TPVB group [chi-square (5) = 34.39, p < .001]. In the SPSIPB group, post hoc analysis (Bonferroni-adjusted alpha = 0.0033) showed VAS scores increased from baseline to peak levels at 1–2 h (0 h vs. 1 h, p < .001; 0 h vs. 2 h, p = 0.001), then declined toward baseline by 4 h and remained stable through 24 h. In the TPVB group, VAS scores increased more gradually, reaching peak levels at 12 h (median 20 mm, +11 mm from baseline) before declining at 24 h (median 18 mm).

For VAS cough scores, a significant change over time was observed in the SPSIPB group [chi-square (5) = 32.62, p < .001] but not in the TPVB group [chi-square (5) = 10.02, p = 0.075].

**Figure 3:**
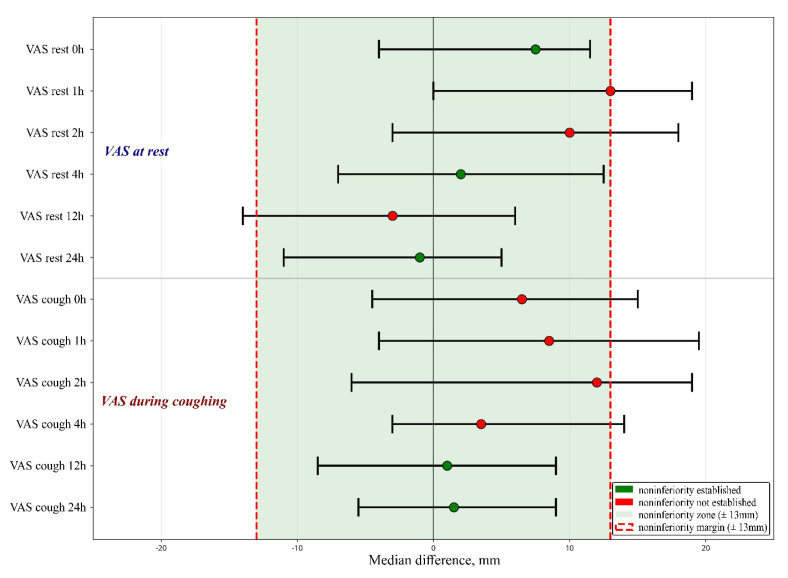
Noninferiority analysis: median differences of VAS scores. VAS: visual analogue scale.

Noninferiority was assessed using the Hodges–Lehmann median difference with 95% bootstrap CI and a prespecified margin of 13 mm (Figure 3). For VAS scores at rest, noninferiority of SPSIPB was demonstrated at three of six time points: 0 h (median difference 7.5 mm, 95% CI: −4 to 11.5), 4 h (median difference 2 mm, 95% CI: −7 to 12.5), and 24 h (median difference −1 mm, 95% CI: −11 to 5). At these time points, the upper bound of the 95% CI did not exceed the 13 mm margin. Noninferiority was not demonstrated at 1 h (95% CI: 0 to 19) or 2 h (95% CI: −3 to 18), as the upper bounds exceeded 13 mm. At 12 h, the 95% CI (−14 to 6) crossed the lower margin of −13 mm.

For VAS scores during coughing, noninferiority was demonstrated at 12 h (95% CI: −8.5 to 9) and 24 h (95% CI: −5.5 to 9). Noninferiority was not demonstrated at 0 h (95% CI: −4.5 to 15), 1 h (95% CI: −4 to 19.5), 2 h (95% CI: −6 to 19), or 4 h (95% CI: −3 to 14), as the upper bounds exceeded 13 mm at each time point.

The 24-h cumulative pain burden, measured by AUC, did not differ between groups for VAS at rest (SPSIPB 426 mm/h vs. TPVB 426.5 mm/h, p = 0.652) or VAS during coughing (SPSIPB 690 mm/h vs. TPVB 664.2 mm/h, p = 0.684; Table 3). The mean pain-reduction slope over 24 h was also comparable (SPSIPB: 1.46 ± 1.94 mm/h; TPVB: 1.29 ± 2.20 mm/h; p = 0.842).

**Figure 4:**
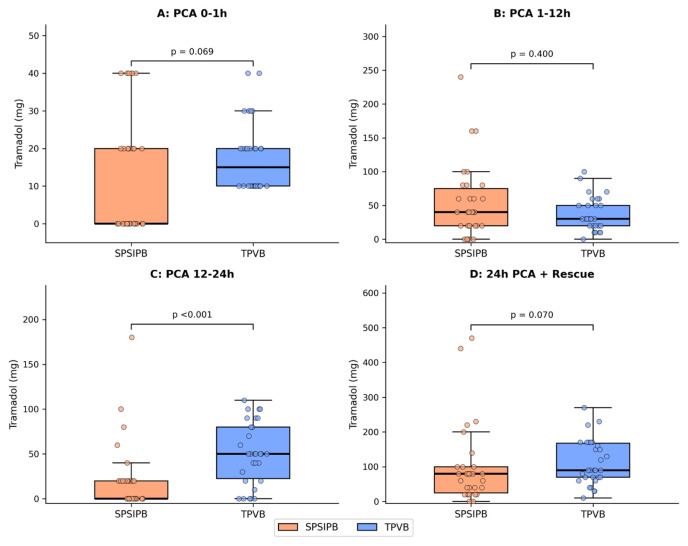
Postoperative opioid consumption during the following time intervals (A: PCA 0–1 h; B: PCA 1–12 h; C: PCA 12–24 h; D: 24h PCA + Rescue). PCA: patient-controlled analgesia, SPSIPB: serratus posterior superior intercostal plane block, TPVB: thoracic paravertebral block.

Postoperative opioid consumption is detailed in Table 4 and Figure 4. In the 0-to-1-h and 1-to-12-h intervals, tramadol consumption did not differ between groups (p = 0.069 and p = 0.400, respectively). In the 12-to-24-h interval, the SPSIPB group consumed significantly less tramadol than the TPVB group [median 0 mg (IQR 0–20) vs. 50 mg (IQR 22.5–80), median difference −30 mg, 95% CI: −50 to −20; p < 0.001]. Total 24-h PCA tramadol consumption did not differ significantly between groups [SPSIPB 80 mg (IQR 25–95) vs. TPVB 90 mg (IQR 62.5–147.5), median difference −30 mg, 95% CI: −60 to 0; p = 0.068]. Total 24-h opioid consumption, including rescue analgesia, was also comparable [SPSIPB 80 mg (IQR 25–100) vs. TPVB 90 mg (IQR 70–167.5), median difference −30 mg, 95% CI: −70 to 0; p = 0.070]. Fewer patients in the SPSIPB group required rescue analgesia (5/30, 16.7%) than in the TPVB group (7/30, 23.3%), though this difference was not statistically significant (p = 0.748).

There were no statistically significant differences in PONV incidence or patient satisfaction scores between groups (p > 0.05; Table 4). No patients in either group experienced pruritus. No block-related complications (pneumothorax, vascular injury, local anesthetic systemic toxicity, or neurological deficit) were observed in either group.

## 4. Discussion

In this randomized noninferiority trial of 60 patients undergoing unilateral mastectomy, we found that SPSIPB was noninferior to TPVB for resting VAS pain scores at 0 h, 4 h, and 24 h. Noninferiority was not demonstrated at 1 h and 2 h, time points at which TPVB provided a clinically meaningful analgesic advantage. At 12 h, the CI crossed the lower boundary, reflecting numerically lower VAS scores in the SPSIPB group rather than inferiority. The 24-h cumulative pain burden measured by AUC was virtually identical between the two techniques (426 mm/h vs. 426.5 mm/h, p = 0.652), indicating comparable overall analgesic efficacy across the full observation period despite the divergent temporal profiles. The most striking secondary finding was the late-phase opioid-sparing effect of SPSIPB. Both techniques were well tolerated, with no block-related complications in either group.

TPVB deposits local anesthetic directly into the paravertebral space, achieving rapid blockade of ventral rami adjacent to spinal nerve roots [1,20,21]. This mechanism accounts for the early analgesic superiority observed in multiple independent trials. Wittayapairoj et al. reported lower numerical rating scale (NRS) scores in the paravertebral group at postanesthesia care unit discharge (2.73 vs. 4.41, p = 0.002) in a double-blind trial of 44 mastectomy patients comparing ESPB with TPVB [22]. Swisher et al. found higher median NRS scores in the ESPB group (3 vs. 0, p = 0.001) and formally rejected noninferiority of ESPB to paravertebral block in 100 patients [7]. Santonastaso et al. confirmed the same pattern with bilevel techniques in 82 patients (NRS at 2 h: TPVB 0.78 vs. ESPB 1.70, p < 0.001), with differences resolving by 12 h [23]. In our trial, the 1-h VAS difference of 13 mm at rest sits at the MCID threshold validated by Gallagher et al. [17], indicating that TPVB’s early advantage, while statistically significant, represents the smallest perceptible clinical difference. By 4 h, the between-group gap had narrowed to 3 mm (95% CI: −7 to 12.5). We believe the rapid onset of TPVB reflects direct ventral rami blockade within the paravertebral space, a mechanism that interfascial-plane blocks cannot replicate as quickly.

The lower tramadol consumption observed with SPSIPB in the 12-to-24-h interval should be interpreted with caution, as the comparator in this trial was TPVB rather than an anterior or interfascial plane block. TPVB delivers local anesthetic into the paravertebral space, a highly vascular compartment that facilitates rapid systemic absorption. Therefore, the apparent late-phase advantage of SPSIPB may reflect earlier bupivacaine washout from the paravertebral space rather than an intrinsically prolonged effect of SPSIPB itself. That said, indirect comparisons with published data on other interfascial plane blocks suggest that late-phase opioid sparing is not a general characteristic of these techniques. Genc et al. found that both ESPB and pectoserratus plane block reduced 24-h morphine consumption compared with control in 90 patients after breast-conserving surgery, yet the absolute reduction remained modest at 2 mg to 3 mg morphine per patient [24]. Bashandy and Abbas reported that PECS I+II block lost its opioid-sparing advantage over control entirely in the 12-to-24-h interval (p = 0.519) despite strong early-phase efficacy in 120 patients [25]. Kulturoglu et al., working at our institution, reported 24-h tramadol consumption of 58.3 mg with rhomboid intercostal block and 68.3 mg with PECS block in 72 patients after modified radical mastectomy; neither block showed a late-period advantage [26]. Cesur et al. reported that bilevel ESPB consumed less morphine than PECS block at all time points in 67 patients, with the greatest separation at 6 h and 12 h (p < 0.001), suggesting that posterior blocks may sustain their analgesic advantage longer than anterior approaches [27]. One anatomical factor that may contribute to the more consistent spread of SPSIPB compared with ESPB is the fascial compartment in which each block is deposited. ESPB is performed deep to the erector spinae muscle group, which consists of the spinalis, longissimus thoracis, and iliocostalis muscles and lacks a well-defined single fascial boundary. Zhang et al. noted that ESPB efficacy depends on individual variations in fascial integrity and connectivity, which may explain the inconsistent paravertebral spread observed in cadaveric and imaging studies [28]. SPSIPB, by contrast, is deposited between the serratus posterior superior muscle, a thin quadrilateral muscle with well-defined fascial boundaries, and the intercostal muscles [11]. This may provide a more predictable interfascial compartment for local anesthetic retention and spread. Whether this anatomical distinction translates into a genuine duration advantage requires direct comparison between SPSIPB and anterior interfascial plane blocks (PECS, ESPB, serratus anterior plane block) within the same trial, which has not been performed to date.

The divergent temporal profiles of SPSIPB and TPVB can be explained by both anatomical and pharmacokinetic differences between the two injection sites. TPVB provided superior early analgesia, while SPSIPB showed a delayed onset with sustained effect. The comparable 24-h AUC values confirm that this temporal redistribution does not alter the total analgesic burden. Pharmacokinetically, TPVB delivers local anesthetic into the paravertebral space, a highly vascular compartment containing intercostal vessels and the sympathetic chain. This achieves rapid blockade but also promotes rapid systemic absorption, thereby limiting the duration of a single injection [20,21]. SPSIPB targets the fascial plane between the serratus posterior superior muscle and the intercostal muscles, a compartment that is relatively avascular compared with the paravertebral space. Local anesthetic dispersion within this interfascial plane is governed by bulk flow and slow diffusion rather than rapid vascular uptake [11,29]. Anatomically, TPVB accesses the paravertebral space with direct blockade of ventral and dorsal rami, whereas SPSIPB lies superficial to the superior costotransverse ligament, relying on fascial spread to reach intercostal nerves and the dorsal ramus. Tulgar et al. confirmed, in cadaveric injections, that SPSIPB dye spread from C7 to T7, with consistent staining of dorsal rami and lateral cutaneous branches [11], and clinical dermatomal mapping in case series demonstrated blockade extending from C3 to T10 [13]. The costotransverse block, anatomically the closest periparavertebral technique to SPSIPB, achieved a 43% reduction in 24-h morphine consumption compared with control in 70 breast surgery patients, with the strongest analgesic effect at 1 h and 3 h [30]. Fusco et al. emphasized that fascial plane block efficacy depends on the proximity of the injection site to target neural structures and the capacity for communication between fascial compartments [29]. The combination of this dual neural target and a pharmacokinetic depot effect in a low-vascularity compartment offers a coherent mechanistic explanation for the sustained analgesic advantage of SPSIPB in the late postoperative period.

From a clinical perspective, the identical 24-h AUC values (426 mm/h vs. 426.5 mm/h) indicate that the overall analgesic burden experienced by patients was the same regardless of block assignment. The difference lies in the temporal distribution: TPVB front-loads its analgesic effect while SPSIPB provides a more gradual onset with sustained late-phase coverage. For enhanced recovery protocols, where patients are discharged with oral analgesics and benefit from reduced late opioid requirements, the analgesic profile of SPSIPB may be particularly well-suited. The late-phase opioid reduction may also carry implications for chronic pain prevention. Gartner et al. reported persistent pain in 47% of breast surgery patients, with acute pain severity as the strongest predictor [2]. Chen et al. demonstrated in a narrative review that regional anesthesia reduces chronic postsurgical pain with a Cochrane odds ratio of 0.61 for paravertebral block [3]. Whether the opioid-sparing effect of SPSIPB translates into reduced chronic pain incidence requires prospective evaluation with follow-up of at least 3 m. An earlier trial from our institution comparing SPSIPB with ESPB also demonstrated lower tramadol consumption in the SPSIPB group (median 60 mg vs. 120 mg, p = 0.044) [16]. Similarly, Dogan et al. observed a concordant temporal pattern in 70 patients after video-assisted thoracoscopic surgery, where TPVB provided lower VAS scores at 1 h (19 mm vs. 26 mm, p = 0.031) with no between-group differences thereafter, and 24-h tramadol consumption numerically favored SPSIPB without reaching statistical significance (150 mg vs. 220 mg, p = 0.129) [18]. The convergence of three trials across two institutions and two surgical populations strengthens the case for a reproducible opioid-sparing effect, although confirmation from independent centers with pharmacokinetic sampling is needed.

The only previous noninferiority trial involving a periparavertebral interfascial plane block and TPVB in breast surgery was conducted by Swisher et al., who rejected noninferiority of ESPB compared with paravertebral block in 100 patients, using a margin of 1.5 NRS points [7]. That result was attributed in part to the inconsistent paravertebral spread of ESPB, which reaches the paravertebral space in fewer than half of injections [8]. Our trial addresses a different clinical question: whether a periparavertebral block targeting the serratus posterior superior intercostal plane, anatomically closer to the paravertebral space than ESPB and without requiring visualization of the transverse process or pleural line [11,31], can achieve noninferior analgesia compared with TPVB. The noninferiority margin of 13 mm was selected a priori based on the MCID for the 100-mm VAS prospectively validated by Gallagher et al. in 96 emergency department patients (95% CI: 10–16 mm) [17]. The 13 mm value represents the median of this validated CI, equidistant between the lower (10 mm) and upper (16 mm) bounds, and thus reflects a balanced, conservative selection within the established range. This threshold was derived from an acute pain population outside the surgical setting, and its direct applicability to postoperative breast surgery pain has not been independently validated. However, a 13-mm threshold is consistent with the general acute pain literature, in which MCID estimates for 100-mm VAS typically range from 9 to 15 mm across diverse clinical settings [17]. Arik et al. and Dogan et al. nonetheless adopted the same 13-mm margin in their SPSIPB trials [16,18], which enables cross-study comparison within this emerging literature. Assay sensitivity was maintained: the TPVB group’s median resting VAS scores (range 9–20 mm) and 24-h tramadol consumption (median 90 mg) are consistent with prior single-injection TPVB data from Swisher et al. [7], Wittayapairoj et al. [22], and Santonastaso et al. [23], confirming that the active comparator performed at its established efficacy level. The zero dropout rate rendered the intention-to-treat and per-protocol populations identical, eliminating the bias toward noninferiority that intention-to-treat analysis can introduce when protocol deviations dilute between-group differences.

Several limitations should be considered when interpreting these results. First, this was a single-center study conducted at a tertiary hospital with a single experienced operator, which may limit generalizability to centers with different levels of ultrasound expertise or different institutional practices. Both SPSIPB and TPVB require familiarity with posterior thoracic sonoanatomy, and block success rates may differ across operators and institutions as experience with SPSIPB accumulates. TPVB in particular carries procedural risks related to its proximity to the pleura and neuraxis [20,21], and its performance in less experienced hands may be associated with a higher rate of technical failures or complications than observed in the present trial. Conversely, the relative technical simplicity of SPSIPB, which does not require visualization of the transverse process or pleural line [31], may confer a reproducibility advantage in lower-volume settings. The single-operator design limits external generalizability but eliminates interoperator variability and ensures that any observed differences in analgesic outcomes reflect the pharmacological and anatomical properties of the two techniques rather than differences in technical execution. Multicenter trials involving operators at varying levels of experience are needed to define the learning curve for SPSIPB and to confirm that the noninferiority result is reproducible across institutions. Second, only the outcome assessor was blinded; patients and the block operator were necessarily aware of the assigned technique. Performance bias cannot be excluded, although the standardized surgical and analgesic protocols, single-operator design, and objective primary outcome (VAS at predefined time points) mitigate this concern. Third, the noninferiority margin of 13 mm, while based on validated MCID data [17], represents the smallest clinically perceptible change; a narrower margin (e.g., 10 mm) would have required a larger sample and might have altered the noninferiority conclusion at specific time points. Fourth, dermatomal sensory assessment was not performed, as all blocks were administered immediately before induction of general anesthesia, precluding cutaneous sensory testing in awake patients. This limits direct correlation between block spread and analgesic outcomes. The extent of SPSIPB coverage in our cohort can only be estimated from existing anatomical and clinical data: cadaveric dye injection by Tulgar et al. demonstrated spread from C7 to T7 with staining of dorsal rami and lateral cutaneous branches [11], and clinical dermatomal mapping in case series confirmed sensory blockade extending from C3 to T10 [13,14]. These reports consistently suggest that SPSIPB provides multilevel thoracic coverage adequate for unilateral mastectomy, but individual variability in fascial plane spread and the absence of prospective sensory mapping in an RCT population remain important limitations. Notably, Dogan et al. reported comparable findings in their SPSIPB vs. TPVB trial in thoracic surgery without dermatomal assessment [18]. Fifth, the optimal local anesthetic volume for SPSIPB has not been established. Bidak et al. showed that increasing ESPB volume from 20 mL to 30 mL did not improve analgesia after mastectomy in 43 patients, suggesting that anatomical plane location may be more important than volume [32]; whether a similar dose–response plateau applies to SPSIPB remains unknown. Future trials should evaluate volume–response relationships for SPSIPB and incorporate preincision sensory mapping under sedation as a standard outcome measure. Sixth, the 24-h follow-up period does not capture the trajectory of analgesic requirements beyond the first postoperative day, nor does it address chronic postsurgical pain outcomes, which affect nearly half of breast surgery patients [2]. Santonastaso et al. reported no chronic pain at 6 m in either the TPVB or ESPB group in their 82-patient trial [23]; a similar long-term assessment would have strengthened our conclusions.

## 5. Conclusion

SPSIPB demonstrated noninferiority to TPVB for postoperative analgesia at the majority of time points following unilateral mastectomy. The distinct temporal analgesic profiles of the two techniques, with TPVB providing early-phase superiority and SPSIPB offering late-phase opioid sparing, give clinicians flexibility in tailoring regional anesthesia to individual patient and surgical priorities. Given the comparable overall analgesic burden and the favorable safety profile of both blocks, SPSIPB warrants evaluation in multicenter trials with extended follow-up to define its role in perioperative breast analgesia.

## Figures and Tables

**Table 1:** Demographic and clinical characteristics.

Characteristic		SPSIPB group (n = 30)	TPVB group (n = 30)	p
Age (y), median (IQR)		65.5 (46–74)	65.5 (52.5–73)	0.515
BMI (kg/m^2^), mean ± SD		24.6 ± 2	25.2 ± 2.1	0.256
ASA Status, n (%)	II	13 (43.3%)	15 (50%)	0.605
	III	17 (56.7%)	15 (50%)	
Comorbidities, n (%)	Yes	15 (50%)	18 (60%)	0.436
Smoking, n (%)	Yes	-	2 (6.7%)	0.492
Axillary dissection, n (%)	Yes	9 (30%)	7 (23.3%)	0.559
Surgery Duration (min), median (IQR)		105 (100–110)	107.5 (95–120)	0.450
Hospital Stay (d), median (IQR)		3 (2.2–4)	4 (3–4)	0.333
Remifentanil (mcg), median (IQR)		404 (278.5–600)	359 (273.8–492.2)	0.564

Data are presented as mean ± SD for normally distributed variables or median (IQR) for nonnormally distributed variables. Categorical variables are presented as n (%). P-values were calculated using the Independent t-test, Mann–Whitney *U* test, chi-square test, or Fisher’s exact test as appropriate.

**Table 2:** VAS scores at rest and during coughing at different time points.

Outcome	SPSIPB group (n = 30)	TPVB group (n = 30)	Median difference (95% CI)	P
**VAS at rest**				
0th h, median (IQR)	16.5 (0–21)	9 (4.2–15.8)	7.5 (−4 to 11.5)	0.139
1st h, median (IQR)	23.0 (12.2–34)	10 (6–22)	13 (0 to 19)	**0.006**
2nd h, median (IQR)	25 (12.5–33.8)	15 (8–25)	10 (−3 to 18)	0.065
4th h, median (IQR)	17.5 (9.5–26.8)	15.5 (1.5–24.8)	2 (−7 to 12.5)	0.273
12th h, median (IQR)	17 (10–26)	20 (12–28)	−3 (−14 to 6)	0.198
24th h, median (IQR)	17 (8.5–19.8)	18 (12–23.8)	−1 (−11 to 5)	0.182
**VAS during coughing**				
0th h, median (IQR)	24.5 (10.5–31.8)	18 (12–24.8)	6.5 (−4.5 to 15)	0.257
1st h, median (IQR)	34.5 (22.2–42.5)	26 (15.2–31.8)	8.5 (−4 to 19.5)	**0.030**
2nd h, median (IQR)	34 (20–43.5)	22 (14.2–37.2)	12 (−6 to 19)	0.113
4th h, median (IQR)	30 (21.2–34.5)	26.5 (11.8–34.8)	3.5 (−3 to 14)	0.239
12th h, median (IQR)	30 (19.8–33.8)	29 (21–33)	1.0 (−8.5 to 9)	0.796
24th h, median (IQR)	27.5 (19.2–33.5)	26 (19.2–30)	1.5 (−5.5 to 9)	0.739

Data are presented as median (interquartile range). P-values were calculated using the Mann–Whitney *U* test. Statistically significant at p < 0.05. All median differences were calculated using the Hodges–Lehmann estimator with 95% bootstrap CIs (1000 iterations). VAS: visual analog scale; SPSIPB: serratus posterior superior intercostal plane block; TPVB: thoracic paravertebral block; IQR: interquartile range; CI: confidence interval.

**Table 3:** Time series analysis of postoperative pain trajectories.

Metric	SPSIPB group (n = 30)	TPVB group (n = 30)	P
AUC rest (mm/h)	426 (236.1–602.9)	426.5 (247.4–628.5)	0.652
AUC cough (mm/h)	690 (507.9–819.2)	664.2 (452.5–799.8)	0.684
Average slope (mm/h)	1.46 ± 1.94	1.29 ± 2.20	0.842

**Table 4:** Postoperative analgesic consumption and clinical outcomes.

Outcome		SPSIPB group (n = 30)	TPVB group (n = 30)	Difference (95% CI)	P
PCA, mg	0–1 h, median (IQR)	0 (0–20)	15 (10–20)	−10 (−10 to 0)	0.069
	1–12 h, median (IQR)	40 (20–75)	30 (20–50)	10 (−10 to 30)	0.400
	12–24 h, median (IQR)	0 (0–20)	50 (22.5–80)	−30 (−50 to −20)	**<.001**
PCA total 24 h (mg), median (IQR)		80 (25–95)	90 (62.5–147.5)	−30 (−60 to 0)	0.068
Total opioid (mg), median (IQR)		80 (25–100)	90 (70–167.5)	−30 (−70 to 0)	0.070
Rescue needed, n (%)		5 (16.7%)	7 (23.3%)	-	0.748
PONV	1, n (%)	25 (83.3%)	24 (80%)	-	0.936
	2, n (%)	4 (13.3%)	5 (16.7%)		
	3, n (%)	1 (3.3%)	1 (3.3%)		
Patient satisfaction	Median (IQR)	5 (4–5)	5 (4–5)	-	0.881
	3, n (%)	3 (10%)	2 (6.7%)		
	4, n (%)	9 (30%)	10 (33.3%)		
	5, n (%)	18 (60%)	18 (60%)		

Data are presented as median (interquartile range) or n (%). P-values were calculated using the Mann–Whitney *U* test, Pearson’s chi-square test, or Fisher’s exact test as appropriate. Median differences calculated using the Hodges–Lehmann estimator with 95% bootstrap CIs (1000 iterations). PCA: patient-controlled analgesia; PONV, postoperative nausea and vomiting; SPSIPB: serratus posterior superior intercostal plane block; TPVB: thoracic paravertebral block; IQR: interquartile range; CI: confidence interval.
